# Use of compost in the uptake mitigation of arsenic in *Beta vulgaris* L. var. *cicla*

**DOI:** 10.1002/jsfa.12026

**Published:** 2022-06-09

**Authors:** Silvia Rita Stazi, Enrica Allevato, Rosita Marabottini, Leonardo Digiesi, Andrea Vannini, Gabriele Chilosi

**Affiliations:** ^1^ Department of Chemical, Pharmaceutical and Agricultural Science (DOCPAS) University of Ferrara Ferrara Italy; ^2^ Department for Innovation in Biological, Agrofood and Forest systems (DIBAF) University of Tuscia Viterbo Italy

**Keywords:** arsenic uptake, bioaccumulation factor, bioavailability, food safety, translocation factor

## Abstract

**Background:**

Arsenic (As) may represent a risk for crop yield quality and human health since it may accumulate in the edible plant organs with the potential of leading to acute or chronic toxic effects in varied segments of the population. Management of soil fertility through compost has proven to be a valuable practice for increasing and maintaining soil organic matter, with nutritional benefits for crops. This work aimed to evaluate Swiss chard yield and the change in the bioavailability, bioaccumulation, and partitioning of As in the response of the use of compost or conventional mineral fertilization in an open‐field trial conducted in a volcanic area in central Italy characterized by the natural contamination of As in soil.

**Results:**

Compost treatment led to a short‐term increase trend in soil organic carbon, total nitrogen, and available phosphorus in a significant way. In the compost‐amended plots, the mitigation of the As uptake was detected in leaves, which are the edible part of Swiss chard. The As bioaccumulation factor in leaves of Swiss chard and the translocation factor for leaves/roots were also decreased using compost.

**Conclusion:**

Fertilization by compost can improve soil fertility, sustain Swiss chard production, and mitigate As accumulation in leaves of this crop grown in a naturally As‐contaminated soil. © 2022 The Authors. *Journal of The Science of Food and Agriculture* published by John Wiley & Sons Ltd on behalf of Society of Chemical Industry.

## INTRODUCTION

Plants can absorb non‐essential elements from contaminated soils depending on their bioavailability as a consequence of the chemical nature and concentration of the elements, soil chemical–physical properties, presence of other ions in the soil, and plant species.^1^, ^2^ Among non‐essential elements, arsenic (As) represents a risk for crop yield quality and human health, since it may accumulate in the edible plant organs with the potential of leading to acute or chronic toxic effects in varied segments of the population.^3^, ^4^ Inorganic As has been classified as a group 1 carcinogenic substance by the International Agency for Research on Cancer.^5^ The As concentration rarely exceeds 10 mg kg^−1^ in uncontaminated soils, but in contaminated soils the inorganic As can reach high concentrations, above 50.0 mg kg^−1^.^6^, ^7^ Based on the risk to human health, the European Commission has introduced maximum allowable levels of inorganic As in rice and rice products.^8^ The European Food Safety Authority's (EFSA's) scientific report on the evaluation of the complete diet showed that the average exposure to inorganic As is below the reference range. However, EFSA recommends that various foods be monitored to assess dietary exposure to inorganic As.^9^, ^10^, ^11^ Exposure to As may be associated with the consumption of different food crops, such as leafy vegetables, which can adsorb As from contaminated soil and accumulate it in their edible parts.^12^, ^13^, ^14^, ^15^

Swiss chard (*Beta vulgaris* subsp. *cicla*) is an important horticultural crop distributed worldwide. In Italy, this crop is cultivated in both greenhouses and open fields, covering approximately an annual average of 5500 ha^16^ and generating an important income for farmers. Swiss chard is considered one of the healthiest vegetables because it contains a large number of nutraceuticals, such as minerals, vitamins, and phytochemicals.^17^, ^18^ Despite these excellent nutraceutical characteristics, Swiss chard is prone to accumulate heavy metals when grown in contaminated soils. In a field study in Spain, Peris *et al*.^19^ reported contents of cadmium and lead in edible parts of lettuce and Swiss chard cultivated in contaminated soil were higher than their tolerance limits in crops. A high accumulation of heavy metals was found in Swiss chard grown in two sites in Ethiopia irrigated with polluted water.^20^ Data from research conducted in the Basque Country (Spain) to investigate the uptake, translocation, and accumulation of several nutrients and non‐essential elements in Swiss chard indicated that they were mainly immobilized in roots, whereas some non‐essentials, such as strontium, barium, and cadmium, were also highly accumulated in leaves.^21^ In a trial where two chard varieties (var. *cicla* and var. *d'ampuis*) were grown in As‐contaminated soil and using contaminated irrigation water, a high level of As accumulation was found in roots and, to a lesser extent, in leaves.^22^

Among the fertilization strategies, management of soil fertility through compost from various feedstocks has proven to be a valuable practice for increasing and maintaining soil organic matter, with nutritional benefits due to the provision of nitrogen and other nutrients and positive effects on yield, health, and quality for many crops.^23^ Incorporating compost or other soil organic amendments can remediate soils by changing the bioavailability of heavy metals.^24^ The immobilization of such contaminants by compost is considerably affected by mineral ions, humic substances, and microbes present in the compost, thus reducing the ecological and environmental risk of heavy metals in agricultural soils.^25^ Therefore, the use of compost can be considered a sustainable fertilization system according to the circular economy approach, with the potential of mitigating the uptake of dangerous non‐essential elements in contaminated soils.^26^

In Italy, the natural geochemical background is highly variable, with several areas of geogenic As.^27^ In the experimental farm of the University of Tuscia, located in the Monti Cimini volcanic district in central Italy, a plot with a high concentration of As in soil was found, presumably as a result of irrigation with water drawn in the past from a geothermal source.^7^, ^28^ Since different crops, including leafy vegetables, are important contributors in Italy to population exposure to As,^29^ the present work aimed to evaluate the change in the bioavailability, bioaccumulation, and partitioning of As in Swiss chard in response to the use of compost in an open‐field trial conducted in the aforementioned contaminated site. Moreover, the influence of compost fertilization on biomass responses was analyzed.

## MATERIALS AND METHODS

### Experimental site and treatments

The trial was conducted in the 2013 cropping seasons at the Experimental Farm of the University of Tuscia, Viterbo (42.420325, 12.075610, 310 m above sea level), in central Italy, and characterized by a soil of volcanic origin, classified as Typic Xerofluvent with high As concentrations related to the deep‐rising fluids of the active geothermal systems.^7^ The preceding crop was durum wheat. Soil, over an area of ~0.5 ha, was tilled in mid‐April. The trial comprised 18 subplots of 12 m^2^ each, leaving corridors 2 m wide to avoid plot disturbance during sampling and land management practices, which were run from the corridors. The experimental design had four treatments, each replicated four times with a random block design. Control plots (C) received no fertilizer. Conventional fertilization plots received only mineral fertilizer (MF) in the form of NPK (Start‐UP NPK 15‐8‐15 + 2MgO + 25SO_3_; Agripoint, Latina, Italy). The compost treatment received about 35 t ha^−1^, a dose capable of maintaining fertility and providing nutrients at the soil site condition for horticultural crops.^30^ Certified compost from urban waste and sewage sludge (ACEA Ambiente, Rome, Italy) with chemical, physical, and biological characteristics according to the Italian law^31^ was used in the present study. Swiss chard (*B. vulgaris* L., var *cicla* (L.); Olter sementi, Milan, Italy) seed was sown in May at 7 kg ha^−1^ in straight lines, with 25 cm of space between each seed and 5 cm between plants in a row (plant density 60 m^−2^). The plots received daily drip irrigation by a single‐line drip irrigation tape with 30 cm spaced emitters laid over the soil surface. Harvesting occurred in July by cutting the aboveground biomass a few centimeters above the soil level.

### Soil and compost analyses

Soil sampling was carried out before sowing the Swiss chard crop. After removing the litter layer, two soil cores (0–20 cm depth) were taken from each plot and then pooled together, for a total of 18 soil samples. The soil samples were air‐dried, sieved (<2 mm), and preserved at room temperature. Then, before biochemical analyses, the soil moisture content of air‐dried samples was adjusted to 60% of their water‐holding capacity, and soils were reconditioned for 5 days.

The main soil physical–chemical characteristics were analyzed by an accredited laboratory (Agri‐bio‐eco Srl, Pomezia, Italy) (Table 1).

**Table 1:** General soil properties

Description	Average value
Sand (%)	65
Loam (%)	17
Clay (%)	18
Texture	Sandy loam
Cation‐exchange capacity (meq/100 g)	22.27
pH	7.5
Electrical conductivity (mS cm^−1^)	0.332

The soil analyses were carried out using methods usually employed internationally, adopted in Italy according to the Italian law.^32^ The physical–chemical characteristics of compost are reported in Table 2.

**Table 2:** Physical and chemical analyses of compost used in this study (±1 standard deviation (SD), *n* = 3)

Description	Average value (±SD)	Italian legislation limit (D.lsg 75/10)
pH	6. 7 ± 0.1	6 ≤ *x* ≤ 8.5
Moisture (%)	21.8 ± 0.7	≤50
Ashes (% d.w.)	48.2 ± 1.2	—
Organic carbon (% d.w.)	26.5 ± 2.6	≥20
Organic nitrogen (% d.w.)	2.3 ± 0.05	—
Total nitrogen (% d.w.)	2.6 ± 0.07	—
Total phosphorus (% d.w.)	1.0 ± 0.05	—
Carbon/nitrogen	11.7	≤25
Humic and fulvic carbon (% d.w.)	16.7 ± 1.7	≥7
Salinity (dS m^−1^)	3.2 ± 0.3	—
Chrome(VI) (mg kg^−1^ d.w.)	<0.010	≤0.5
Cadmium (mg kg^−1^ d.w.)	<0.001	≤1.5
Mercury (mg kg^−1^ d.w.)	<0.010	≤1.5
Nichel (mg kg^−1^ d.w.)	7.8 ± 0.8	≤100
Lead (mg kg^−1^ d.w.)	26.3 ± 2.1	≤140
Zinc (mg kg^−1^ d.w.)	242.7 ± 12.1	≤500
Copper (mg kg^−1^ d.w.)	139.1 ± 11.1	≤230

d.w., dry weight.

### Plant sampling

In each plot, whole chard plants (roots included) were sampled in a sub‐portion (1 m^2^) of the plot to evaluate the effect of fertilization on bioavailability, bioaccumulation, and partitioning of As and plant biomass. Sampled plants were rinsed with tap water to remove soil residuals, and then plants were divided into roots and shoots and subjected to analyses.

### Arsenic analyses in soil and plant

The total amount of As in the soil and plant was determined as described by Stazi and co‐workers^33^, ^34^ after mineralization of the samples with concentrated (36%) hydrochloric acid, 69% nitric acid, and 30% hydrogen peroxide (Merck, Darmstadt, Germany) with microwave‐assisted digestion (Mars plus CEM, Cologno al Serio, Italy). For measuring the concentration of the elements we used an inductively coupled plasma optical emission spectrometer (Optima 8000DV; Perkin Elmer Corp., Norwalk, CT, USA) equipped with a Scott nebulizer for soil samples and an ultrasonic nebulizer (USN; Perkin Elmer Corp.) for plant samples. The analysis was performed in triplicate. As_2_O_3_ standard was purchased from CaPurAn (CPA chem, Bogomilovo, Bulgaria), Multielement Standard Solution 6 for inductively coupled plasma was purchased from Merck. The purity of the plasma torch argon was greater than 99.99%. The accuracy of the measurements was assessed using trace metals loamy sand 3 standard reference materials (CRM034; Fluka) and standard leaves (SRM 1573a tomato leaves).

The As mobility in soil was determined as the concentration of the bioavailable element, a parameter influencing plant metal uptake of the element, which is defined by the chemical properties of the metal cations and by the physicochemical characteristics of the soil.^35^ According to Marabottini *et al*.,^36^ to quantify bioavailable As, three soil fractions were studied: water soluble (WS), not specifically sorbed (NSS), and specifically sorbed (SS). The concentration of each bioavailable element is given by the sum of the individual concentration with which the specific element appears in each fraction: WS + NSS + SS. The amount of bioavailable As was used to assess the mobility of the toxic element in soil.

The ability of Swiss chard to translocate As from the roots to the aerial part of the plant was evaluated by the translocation factor (TF),^37^ using the formula TF = *C*_shoot_/*C*_root_, where *C* is the concentration of As.

The plant/soil bioaccumulation factor (BAF) of the specific element (determined as the quotient of the content of heavy metal of the plant to its content of the soil) defines the ability of the plant to accumulate heavy metals^38^ and was calculated as the ratio between the concentration of the total element in leaves and the concentration of the bioavailable element in soil.^37^

### Statistical data analysis

Data were subjected to one‐way and two‐way analysis of variance. Tukey's *post hoc* test was employed to identify pairs of results with significantly different means. Both analysis of variance and a *post hoc* test were performed using the GraphPad Prism computer package (GraphPad Software, San Diego, CA, USA).

The As concentration, its bioavailability in soil, and its accumulation in the root and leaves were subjected to principal component analysis (PCA) to verify the interaction between the different factors able to synthesize the considered variables. All calculations were performed using Excel (Microsoft Corporation, Redmond, WA, USA) and JMP 11.0 statistical software package (SAS Institute, Cary, NC, USA).

## RESULTS

### Soil quality and As concentration and bioavailability

The results at the end of the trial showed that compost treatment led to a short‐term increase trend in soil organic carbon (SOC), total nitrogen (TN), and available phosphorus (P) in a significant way (Table 3). Electrical conductivity (EC) had a significant increase following the application of both compost and MF, whereas the cation exchange capacity (CEC) did not vary compared with the untreated control.

**Table 3:** Soil chemical and physical parameters upon mineral fertilization (MF), compost application, and unfertilized control

Description	Control	MF	Compost
pH	7.5	7.6	7.6
SOC (%)	1.8	1.9	3.3
TN (%)	0.1	0.1	0.2
C/N	11.0	11.0	9.5
Available P (P_2_O_5_) (mg kg^−1^)	17.98^b^	23.29^b^	62.24^a^
EC (mS cm^−1^)	0.3^b^	1.0^a^	1.2^a^
CEC (meq)	22.3	24.1	25.0

Values belonging to the same parameter with different letters are statistically different according to least significant difference (0.05).

SOC, total organic carbon; TN, total nitrogen; C/N ratio, total organic carbon (%)/total organic nitrogen (%); CEC, cation‐exchange capacity; EC, electrical conductivity.

The concentrations of total As and its bioavailability in soil from unfertilized control, MF, and compost treatment are shown in Table 4. Treatment with compost was reflected by a significantly lower As concentration in soil compared with control and MF. By contrast, in compost‐amended plots, a significantly higher As bioavailability percentage was detected than found in MF and unfertilized control.

**Table 4:** Arsenic concentration in soil and its bioavailability upon mineral fertilization (MF), compost application and unfertilized control

Parameter	Control	MF	Compost
Soil concentration (mg kg^−1^)	55.65^a^	52.35^a^	40.26^b^
Bioavailability (%)	15.35^b^	16.95^ab^	18.60^a^

The values are from the average of 12 replicates. Values belonging to the same parameter with different letters are statistically different according to least significant difference (0.05).

### Uptake, partitioning, BAF, and TF of As in plant organs

Our results show that the uptake of As in the root system of Swiss chard was significantly increased by MF in respect of unfertilized control and compost application (Table 5). The highest absorption value of As in the foliar apparatus, which represents the edible part of the plant, was found in plants from unfertilized control, whereas a consistent reduction was recorded in the leaves of plants grown in soil treated with MF and especially with compost, with a respectively 2.8‑ and 4.4‐fold decrease.

**Table 5:** Effect of chemical and compost fertilization on arsenic uptake, partitioning, bioaccumulation factor (BAF) in roots and leaves of Swiss chard, and translocation factor (TF) leaves/roots

Parameter	Control	MF	Compost
Roots uptake (mg kg^−1^)	6.14^b^	11.76^a^	7.74^b^
Leaves uptake (mg kg^−1^)	7.19^a^	2.53^b^	1.62^b^
BAF roots	0.72^b^	1.33^a^	1.04^ab^
BAF leaves	0.84^a^	0.29^b^	0.22^b^
TF leaves/roots	2.17^a^	0.22^b^	0.21^b^

Values belonging to the same parameter with different letters are statistically different according to least significant difference (0.05).

MF, mineral fertilization.

The BAF of As in plant roots and leaves is reported in Table 5. The BAF in the root system was significantly higher in plants grown in soil treated with MF and, to a lesser extent, with compost. By contrast, both MF and compost application resulted in a significant decrease in As TF values in respect of unfertilized control (Table 5).

### Effect of fertilization on crop yield

**Figure 1:**
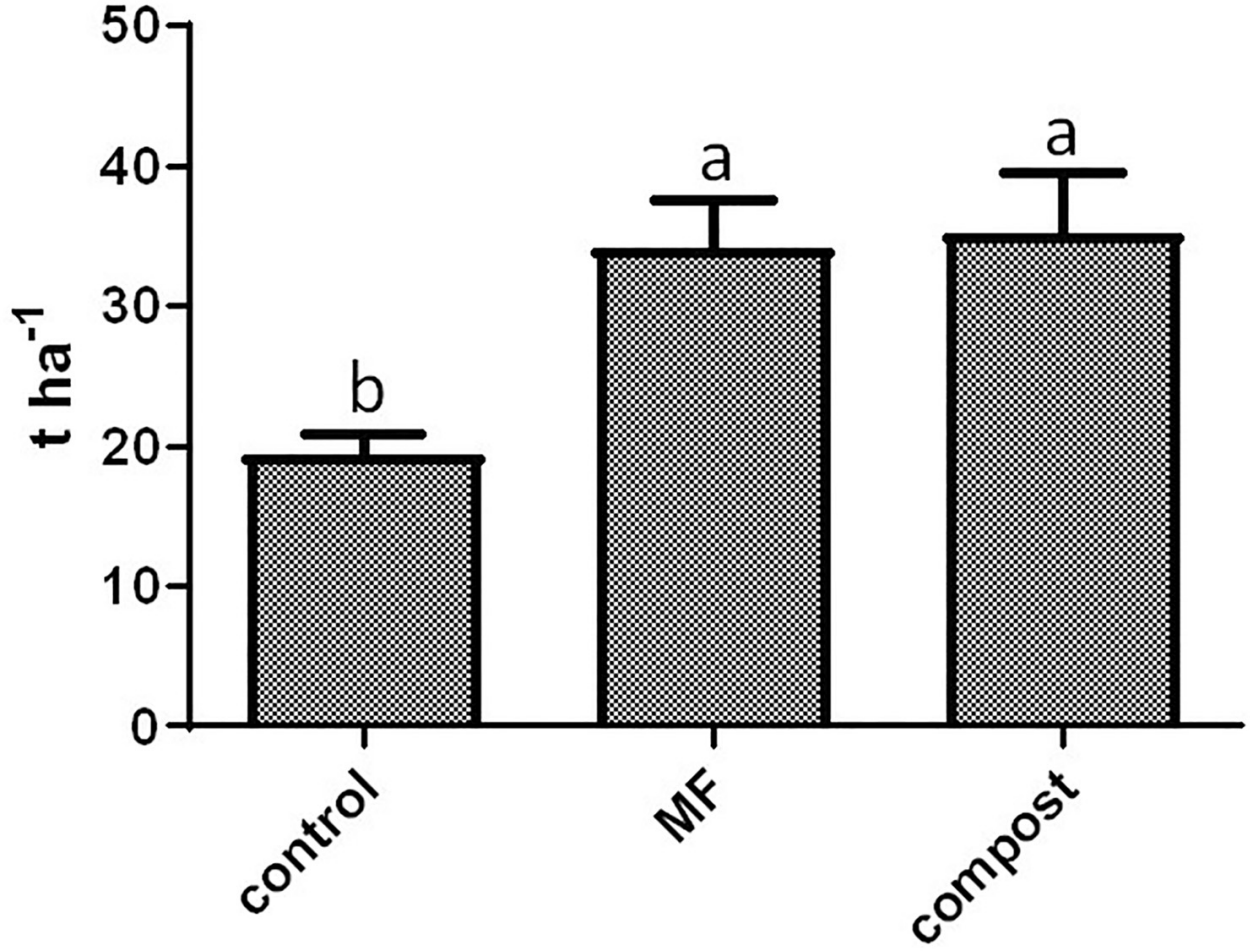
Estimation of Swiss chard yield (t ha^−1^) upon mineral fertilization (MF), compost application, and unfertilized control. Values belonging to the same parameter with different letters are statistically different according to least significant difference (0.05).

Swiss chard yield was expressed as an estimate of the leaf biomass per hectare per each fertilization treatment (Fig. 1). The yield was significantly higher from plants harvested from plots treated with MF and compost compared with the unfertilized control.

### Analysis of the principal components

**Figure 2:**
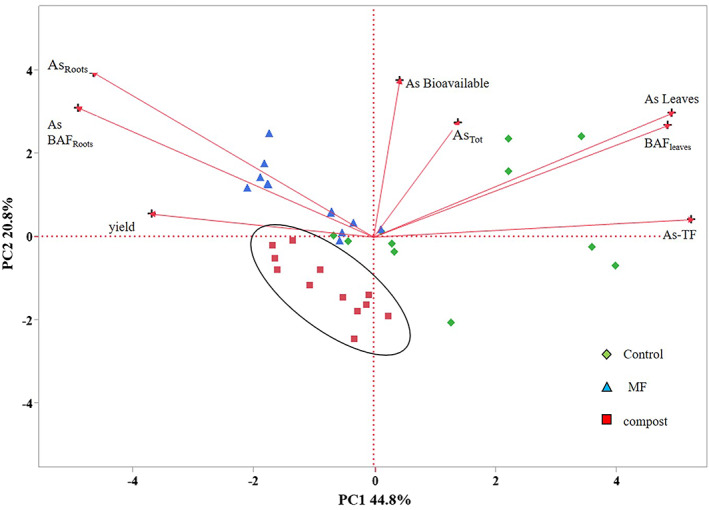
Principal component analysis scatterplot based on total and bioavailable arsenic (As) concentrations in soil and root and leaves of Swiss chard as a function of managed soils. Solid, line group composted soils. PC1 × PC2 explains 65.6% of the total variance. BAF, bioaccumulation factor; DW, dry weight; TF, translocation factor.

PCA of variables was performed on the unfertilized, compost‐amended, and minerally fertilized soils (Fig. 2). The PCA showed that the first and second components explained 65.6% of the total variance.

The score plot indicates that the samples could be divided into three groups: compost amended, mineral fertilized, and not fertilized. This suggests that different management strategies had a strong effect on the behavior of As. The load diagram of the variables indicates that, in particular, the treatment with MF influences the radical absorption of toxic elements (second quadrant) and its BAF. The transport of the toxic element in the leaves explained in the first quadrant highlights the natural tendency of As to be mobile in the soil–plant system.

## DISCUSSION

The progressive decline of organic matter and organic carbon in European cropland is an increasing problem undermining soil fertility and crop productivity.^39^ A wide range of studies focusing on compost use has proven that compost strongly increases soil fertility and crop performance under different types of soil.^40^ In the present work, we confirm that the use of compost applied to the soil of volcanic origin is capable of improving soil‐fertility‐related parameters, such as SOC and available P. However, both MF and compost addition significantly enhanced soil EC, even if well below the Swiss chard salt tolerance threshold.^41^ Moreover, in terms of biomass, compost application led to a level of plant production comparable to that obtained with the use of MF, confirming the potential of compost in plant nutrition according to a circular economy model.

Our results show that the soil where the trial was performed was characterized by high As contamination, confirming previous findings.^7^ We observed that the application of compost caused a significant increase in bioavailability of As in plots fertilized with compost compared with the control, which might be due both to the increase in SOC and the sharp increase in available phosphate. Organic carbon, probably competing with As for sorption sites, such as iron oxides, increases As mobility.^42^

Phosphate, owing to its structural similarity, competes with sites that retain arsenate and promotes As mobility in the soil.^43^

This result is in agreement with those reported by Stazi *et al*.^7^ in a study concerned with the effects of organic *versus* conventional management on soil quality and tomato yield in the area naturally contaminated by As where the present trial was located. They pointed out that an increase in phosphate assimilated into the soil led to greater bioavailability of As. In that case, they had a greater amount of phosphate in the soil treated conventionally. Thus, even if organic management based on green manure increased the total As concentration in the soil, there was a higher bioavailability of As after conventional (12.3%) *versus* organic (9.5%) treatment. Our results confirm that the increased availability of phosphate was responsible for the increase in As bioavailability in compost‐treated soil where P was higher. The addition of phosphate promotes the mobility of As in the soil. In our work, the bioavailability of As after the two treatments does not show significant differences between MF soil and composted soil. However, after the treatments, the phosphate provided by the compost is about threefold that of the conventional one. The greater amount of phosphate available might have promoted the increase in the selectivity of P absorption with respect to As since they are both taken up by the phosphate transporters owing to their structural similarity. This can explain the greater accumulation of As in the roots of plants grown in MF compared with those cultivated in compost‐amended plots. In fact, roots in MF plots were less selective, and took up more arsenate, whereas compost amendment mitigated the absorption of As. It is also important to consider the effect of pH on the mobility of As in the soil. It was observed that, as the pH of the soil increases, the hydroxyl ions replace the As oxyanions in the absorption sites of the soil, leading to their subsequent release in solution, thus increasing the bioavailability of As in the soil.^44^

Apart from some exceptions, plants take up and accumulate heavy metals mainly in the root and secondarily in the aerial part when grown on polluted soils.^45^, ^46^ As shown by the PCA, the different treatments of soils affected the fate of As in the soil–plant system and its bioavailability and phytoavailability. The treatment with compost led to important mitigation of the As uptake, especially in the edible leaves of Swiss chard, and, therefore, for the food chain risk.

This trend was confirmed by the BAF ratio, thus indicating the low phytoavailability of As in plants grown in compost‐treated soil. This scenario was also confirmed by TF ratios, which showed that the treatment with compost mitigated the transport of As from root to leaf with a decrease of tenfold compared with the unfertilized control. Compost and MF application may cause physiological changes in plants as well as changes in soil physical properties and plant nutrients regime, all of which influence As uptake in crops. The BAF indicates the As enrichment in plant biomass, and this ratio is also used to compare the accumulation efficiency of different metals in the soil–plant system.^47^, ^48^ The BAF results indicate a change in As mobility from soil to leaves due to the application of either MF or compost, whereas TF values following both MF and compost application show that As showed a significant decrease in translocation from roots to leaves with respect to the unfertilized control.

## CONCLUSION

The use of compost is capable of increasing soil fertility and supporting the production of crops according to the circular economy model. The results of this work show that the use of compost can mitigate in naturally contaminated soils the absorption of As, confirming that this fertilizer represents an important resource in agroecological and organic farming systems. Therefore, in conditions of a high degree of natural As contamination, the use of compost could be a useful resource for the cultivation of food in otherwise unusable soils. Further research on the long‐term effect of compost on soil As mobility and Swiss chard and other crops’ intake will be necessary.

## References

[1] Peralta‐Videa JR , Lopez ML , Narayan M , Saupe G and Gardea‐Torresdey J , The biochemistry of environmental heavy metal uptake by plants: implications for the food chain. Int J Biochem Cell Biol 41:1665–1677 (2009). 19433308 10.1016/j.biocel.2009.03.005

[2] Allevato E , Stazi SR , Marabottini R and D'Annibale A , Mechanisms of arsenic assimilation by plants and countermeasures to attenuate its accumulation in crops other than rice. Ecotoxicol Environ Saf 185:109701 (2019). 10.1016/j.ecoenv.2019.109701. 31562999

[3] Beccaloni E , Vanni F , Beccaloni M and Carere M , Concentrations of arsenic, cadmium, lead and zinc in homegrown vegetables and fruits: estimated intake by population in an industrialized area of Sardinia, Italy. Microchem J 107:190–195 (2013).

[4] Corradini F , Correa A , Moyano MS , Sepúlveda P and Quiroz C , Nitrate, arsenic, cadmium, and lead concentrations in leafy vegetables: expected average values for productive regions of Chile. Arch Agron Soil Sci 64:299–317 (2018).

[5] IARC Working Group on the Evaluation of Carcinogenic Risks to Humans , Some Drinking‐Water Disinfectants and Contaminants, Including Arsenic, Vol. 84. World Health Organization, International Agency for Research on Cancer IARC Monograph, Lyon, France (2004). PMC7682301 15645577

[6] Mahimairaja S , Bolan NS , Adriano DC and Robinson B , Arsenic contamination and its risk management in complex environmental settings. Adv Agron 86:1–82 (2005).

[7] Stazi SR , Mancinelli R , Marabottini R , Allevato E , Radicetti E , Campiglia E *et al*., Influence of organic management on as bioavailability: soil quality and tomato as uptake. Chemosphere 211:352–359 (2018). 30077931 10.1016/j.chemosphere.2018.07.187

[8] The European Commission , Commission Regulation (EU) 2015/1006 of 25 June 2015 amending regulation (EC) No. 1881/2006 as regards maximum levels of inorganic arsenic in foodstuff. Off J Eur Union Legis 161:14–15 (2015).

[9] EFSA Panel on Contaminants in the Food Chain (CONTAM) , Scientific opinion on arsenic in food. EFSA J 7:1351 (2009).

[10] European Food Safety Authority , Dietary exposure to inorganic arsenic in the European population. EFSA J 12:3597 (2014). 10.2903/j.efsa.2021.6380 PMC7845508 33537067

[11] European Food Safety Authority (EFSA) , Arcella D , Cascio C and Ruiz JÁG , Chronic dietary exposure to inorganic arsenic. EFSA J 19:e06380 (2021). 33537067 10.2903/j.efsa.2021.6380 PMC7845508

[12] Augustsson A , Uddh‐Soderberg T , Filipsson M , Helmfrid I , Berglund M , Karlsson H *et al*., Challenges in assessing the health risks of consuming vegetables in metal‐contaminated environments. Environ Int 113:269–280 (2018). 29157867 10.1016/j.envint.2017.10.002

[13] Ma L , Yang ZG , Kong Q and Wang L , Extraction and determination of arsenic species in leafy vegetables: method development and application. Food Chem 217:524–530 (2017). 27664668 10.1016/j.foodchem.2016.09.015

[14] Qin J , Niu A , Liu Y and Lin C , Arsenic in leafy vegetable plants grown on mine water‐contaminated soils: uptake, human health risk and remedial effects of biochar. J Hazard Mater 402:123488 (2021). 10.1016/j.jhazmat.2020.123488. 32738781

[15] Zheng X , Zhang Z , Chen J , Liang H , Chen X , Qin Y *et al*., Comparative evaluation of *in vivo* relative bioavailability and *in vitro* bioaccessibility of arsenic in leafy vegetables and its implication in human exposure assessment. J Hazard Mater 423:126909 (2022). 10.1016/j.jhazmat.2021.126909. 34454790

[16] Istituto Nazionale Di Statistica , Italy Agricoltura (2020) Available: https://www.istat.it/it/agricoltura?dati [28 April 2021].

[17] Trifunović S , Topalović A , Knežević M and Vajs V , Free radicals and antioxidants: antioxidative and other properties of Swiss chard (*Beta vulgaris* L. subsp. *cicla*). Agric For 61:73–92 (2015).

[18] Mzoughi Z , Chahdoura H , Chakroun Y , Cámara M , Fernández‐Ruiz V , Morales P *et al*., Wild edible Swiss chard leaves (*Beta vulgaris* L. var. *cicla*): nutritional, phytochemical composition and biological activities. Food Res Int 119:612–621 (2019). 30884696 10.1016/j.foodres.2018.10.039

[19] Peris M , Micó C , Recatalá L , Sánchez R and Sánchez J , Heavy metal contents in horticultural crops of a representative area of the European Mediterranean region. Sci Total Environ 378:42–48 (2007). 17306337 10.1016/j.scitotenv.2007.01.030

[20] Gezahegn WW , Srinivasulu A , Aruna B , Banerjee S , Sudarshan M , Narayana PL *et al*., Study of heavy metals accumulation in leafy vegetables of Ethiopia. IOSR J Environ Sci Toxicol Food Technol 11:57–68 (2017).

[21] Liñero O , Cidad M , Carrero JA , Nguyen C and de Diego A , Partitioning of nutrients and non‐essential elements in Swiss chards cultivated in open‐air plots. J Food Compos Anal 59:179–187 (2017).

[22] Yañez LM , Alfaro JA and Mitre GB , Absorption of arsenic from soil and water by two chard (*Beta vulgaris L*.) varieties: a potential risk to human health. J Environ Manage 218:23–30 (2018). 29665483 10.1016/j.jenvman.2018.04.048

[23] Chilosi G , Aleandri MP , Bruni N , Tomassini A , Torresi V , Muganu M *et al*., Assessment of suitability and suppressiveness of on‐farm green compost as a substitute of peat in the production of lavender plants. Biocontrol Sci Technol 27:539–555 (2017).

[24] Bolan N , Kunhikrishnan A , Thangarajan R , Kumpiene J , Park J , Makino T *et al*., Remediation of heavy metal(loid)s contaminated soils – to mobilize or to immobilize? J Hazard Mater 266:141–166 (2014). 24394669 10.1016/j.jhazmat.2013.12.018

[25] Huang M , Zhu Y , Li Z , Huang B , Luo N , Liu C *et al*., Compost as a soil amendment to remediate heavy metal‐contaminated agricultural soil: mechanisms, efficacy, problems, and strategies. Water Air Soil Pollut 227:359 (2016).

[26] Farrell M , Perkins WT , Hobbs PJ , Griffith GW and Jones DL , Migration of heavy metals in soil as influenced by compost amendments. Environ Pollut 158:55–64 (2010). 19773103 10.1016/j.envpol.2009.08.027

[27] Cubadda F , D'Amato M , Aureli F , Raggi A and Mantovani A , Dietary exposure of the Italian population to inorganic arsenic: the 2012–2014 Total Diet Study. Food Chem Toxicol 98:148–158 (2016). 27756704 10.1016/j.fct.2016.10.015

[28] Mancinelli R , Radicetti E , Muleo R , Marinari S , Bravo I and Papetti P , Can hairy vetch cover crop affect arsenic accumulation in vegetable crops? Agriculture 9:89 (2019).

[29] Cubadda F , D'Amato M , Mancini FR , Aureli F , Raggi A , Busani L *et al*., Assessing human exposure to inorganic arsenic in high‐arsenic areas of Latium: a biomonitoring study integrated with indicators of dietary intake. Ann Ig 27:39–51 (2015). 25748504 10.7416/ai.2015.2021

[30] Centemero M , Il ruolo del compost nei piani di fertilizzazione. L'Informatore Agrario 40:57–60 (2002).

[31] Decreto legislativo 29 aprile 2010 , n. 75: Riordino e revisione della disciplina in materia di fertilizzanti, a norma dell'Articolo 13 della legge 7 luglio 2009, n. 88, Gazzetta Ufficiale della Repubblica Italiana 121 N. 106/L (2010).

[32] Ministero per le Politiche Agricole , Metodi ufficiali di analisi chimica del suolo. Decreto Ministeriale 13 settembre 1999. Gazzetta Ufficiale della Repubblica Italiana 248 (1999).

[33] Stazi SR , Mancinelli R , Allevato E , Marabottini R , Campiglia E and Marinari S , Phytoavailability of geogenic arsenic and its partitioning in soil: a case of study in a thermal area of central Italy. EQA Int J Environ Qual 20:27–34 (2016).

[34] Stazi SR , Cassaniti C , Marabottini R , Giuffrida F and Leonardi C , Arsenic uptake and partitioning in grafted tomato plants. Hortic Environ Biotechnol 57:241–247 (2016).

[35] Cuypers A , Remans T , Weyens N , Colpaert J , Vassilev A and Vangronsveld J , Soil–plant relationships of heavy metals and metalloids, in Heavy Metals in Soils, ed. by Alloway BJ . Springer, Dordrecht, Netherlands, pp. 161–193 (2013).

[36] Marabottini R , Stazi SR , Papp R , Grego S and Moscatelli MC , Mobility and distribution of arsenic in contaminated mine soils and its effects on the microbial pool. Ecotoxicol Environ Saf 96:147–153 (2013). 23856118 10.1016/j.ecoenv.2013.06.016

[37] Allevato E , Mauro RP , Stazi SR , Marabottini R , Leonardi C , Ierna A *et al*., Arsenic accumulation in grafted melon plants: role of rootstock in modulating root‐to‐shoot translocation and physiological response. Agronomy 9:828 (2019).

[38] Pachura P , Ociepa‐Kubicka A and Skowron‐Grabowska B , Assessment of the availability of heavy metals to plants based on the translocation index and the bioaccumulation factor. Desalin Water Treat 57:1469–1477 (2016).

[39] Gobin A , Campling P , Janssen L , Desmet N , van Delden H , Hurkens J *et al*., Soil Organic Matter Management across the EU – Best Practices, Constraints and Trade‐Offs. Publications Office, Luxembourg (2011). 10.2779/17252.

[40] Manirakiza N and Şeker C , Effects of compost and biochar amendments on soil fertility and crop growth in a calcareous soil. J Plant Nutr 43:3002–3019 (2020).

[41] Grieve CM , Grattan SR and Maas EV , Plant salt tolerance. Agricultural Salinity Assessment and Management Wallender WW , Tanji KK , 2nd edition, ASCE Manual and Reports on Engineering Practice No. 71 ASCE, Reston, VA, USA: 405–459 (2012).

[42] Redman AD , Macalady DL and Ahmann D , Natural organic matter affects arsenic speciation and sorption onto hematite. Environ Sci Technol 36:2889–2896 (2002). 12144264 10.1021/es0112801

[43] Cao X , Ma LQ and Shiralipour A , Effects of compost and phosphate amendments on arsenic mobility in soils and arsenic uptake by the hyperaccumulator *Pteris vittate* L. Environ Pollut 126:157–167 (2003). 12927487 10.1016/s0269-7491(03)00208-2

[44] Carbonell‐Barrachina A , Jugsujinda A , DeLaune RD , Patrick WH Jr , Burló F , Sirisukhodom S *et al*., The influence of redox chemistry and pH on chemically active forms of arsenic in sewage sludge‐amended soil. Environ Int 25:613–618 (1999).

[45] Angelova V , Ivanova R and Ivanov K , Heavy metal accumulation and distribution in oil crops. Commun Soil Sci Plant Anal 35:2551–2566 (2004).

[46] Kabata‐Pendias A , Trace elements in plants, in Trace Elements in Soils and Plants, 4th edn. CRC Press/Taylor & Francis Group, Boca Raton, FL, USA, pp. 93–122 (2010).

[47] Dradrach A , Karczewska A and Szopka K , Arsenic accumulation by red fescue (*Festuca rubra*) growing in mine affected soils – findings from the field and greenhouse studies. Chemosphere 248:126045 (2020). 32050316 10.1016/j.chemosphere.2020.126045

[48] Salinitro M , Montanari S , Simoni A , Ciavatta C and Tassoni A , Trace metal accumulation and phytoremediation potential of four crop plants cultivated on pure sewage sludge. Agronomy 11:2456 (2021).

